# Changes in anterior segment optical coherence tomography following pars plana vitrectomy without tamponade

**DOI:** 10.1186/s40942-021-00285-w

**Published:** 2021-03-01

**Authors:** Alireza Khodabande, Massood Mohammadi, Hamid Riazi-Esfahani, Shahab Karami, Massood Mirghorbani, Bobeck S. Modjtahedi

**Affiliations:** 1 Department of Ophthalmology, Farabi Eye Research Center, Farabi Eye Hospital, Tehran University of Medical Sciences, Qazvin square, South Karegar Street, Tehran, Iran; 2 Department of Ophthalmology, Southern California Permanente Medical Group, Baldwin Park, CA USA; 3 Department of Research and Evaluation, Southern California Permanente Medical Group, Pasadena, CA USA; 4 Eye Monitoring Center, Kaiser Permanente Southern California, Baldwin Park, CA USA

**Keywords:** Angle, Anterior chamber, Morphology, Optical coherence tomography, Pars plana vitrectomy

## Abstract

**Background:**

To evaluate changes in anterior segment morphology on anterior segment optical coherence tomography (AS-OCT) following pars plana vitrectomy (PPV) without tamponade.

**Methods:**

Patients who underwent PPV without tamponade for epiretinal membrane were evaluated. Eligible patients underwent intraocular pressure (IOP) measurement and AS-OCT preoperatively as well as 1 month and 6 months post-operatively. Anterior chamber width (ACW), anterior chamber depth (ACD), trabecular iris angle (TIA), angle opening distance at 500 and 750 µm (AOD), and trabecular iris space area at 500 and 750 µm (TISA) at four quadrants were recorded and analyzed. Additionally, the mean values of TIA (MTIA), AOD (MAOD), and TISA (MTISA) for each eye (mean of four quadrants) were analyzed.

**Results:**

23 patients completed the study. The mean age of participants was 56.4 ± 3.6 years of age and 13/23 (56%) were female. Mean IOP of patients was 18.1 ± 1.1, 18.3 ± 1.1, and 18.1 ± 1.2 preoperatively,1 month post-operatively, and 6 months post-operatively, respectively. (p = 0.83). No difference was detected post-operatively in measurements of ACW, ACD, MTIA, MAOD500, MAOD750, MTISA500, and MTISA750.

**Conclusion:**

Pars plana vitrectomy without tamponade was not associated with changes in anterior chamber morphology.

## Introduction

Increases in intraocular pressure (IOP) after PPV are commonly observed and occur in 18–28% of patients [1–3]. Elevations in IOP following PPV can be due to angle closure (approximately 20% of cases) via pupillary block, ciliary block, or anterior synechia or open-angle (approximately 80% of cases) including tamponade (especially silicone oil) migration into the anterior chamber, gas or oil overfill, steroid response, and post-op hemorrhage or inflammation [4]. Proposed risk factors for post-PPV IOP elevations include a history of glaucoma [5], diabetes mellitus [6], scleral buckling (SB) [7], lensectomy [8], and the use of silicone oil or expansile gas [7, 9].

It is unclear whether PPV results in significant changes in anterior segment morphology that could pre-dispose patients for IOP elevations. Although prior studies have investigated anterior segment changes after PPV [10–15] most have utilized ultrasound biomicroscopy (UBM) for anterior angle evaluation [10, 11, 14]. UBM provides detailed evaluations of the anterior and posterior segment; however, it is highly operator dependent which creates the risk of intra or inter-observer errors [16]. In contrast, anterior segment optical coherence tomography (AS-OCT) provides a highly detailed AC angle imaging with a resolution of 15 µm (more than 50 µm of UBM) and is less operator dependent [16]. This study sought to characterize changes in anterior segment morphology on AS-OCT after PPV where there was no tamponade utilized and no associated procedures such as scleral buckling or phacoemulsification.

## Methods

This study was conducted at Farabi Eye Hospital, Tehran University of Medical Sciences, a tertiary ophthalmology center in Iran. The study protocol was approved by the Institutional Review Board of the Farabi Eye Hospital, and the study adhered to the Declaration of Helsinki. Eligible patients participated after providing written informed consent.

### Subjects

Patients greater than 18 years of age who underwent PPV for epiretinal membranes between April 2018 to April 2019 were included in this study if there were no secondary procedures or intraocular tamponade used at the time of surgery. Patients were excluded if they had a history of prior intraocular surgery (including phacoemulsification), uveitis, glaucoma, diabetes mellitus, IOP of ≥ 22 mmHg, anterior segment laser therapy, and the use of any topical or systemic drugs that might affect the pupil or accommodation. Also, patients with high myopia (SE ≥ − 6D) or significant hyperopia (SE ≥  + 4D) were excluded.

Preoperative examinations were performed within 7 days before surgery by a glaucoma specialist and included slit-lamp bio microscopy of anterior chamber, Goldmann applanation tonometry, and gonioscopy.

### Image acquisition

Patients underwent anterior segment optical coherence tomography (AS-OCT) preoperatively, 1 month post-operatively, and 6 months post-operatively. AS-OCT (Visante OCT; Carl Zeiss Meditec, Dublin, CA, USA) was performed for all patients in a standard dark room (< 1 lx illumination by digital light meter; Easy View model EA30; Extech Instruments, Inc., Waltham, MA) by a single examiner. Before image acquisition, patients were allowed 5 min for dark adaptation. Scans were centered on the undilated pupil, along the horizontal axis such that the corneal vertex reflex could be observed clearly. Irides of the temporal and nasal quadrants were aligned on a horizontal level by adjusting fixation angle. Raw image scanning in the “anterior segment single mode” (816 × 638 pixels exported image) was used to acquire an image. To ensure non-accommodative status of the tested eye, the patient's distance refraction was used to adjust the fixation target. At least 3 consecutive images were captured, and the image with the best quality of alignment and visibility of the corneal vertex reflex, left scleral spur (LSS)—right scleral spur (RSS) in horizontal scan, and inferior scleral spur (ISS)—superior scleral spur (SSS) in vertical scan was chosen for analysis. The same protocol was followed at each imaging visit for all 23 patients.

### Image analysis

**Fig. 1:**
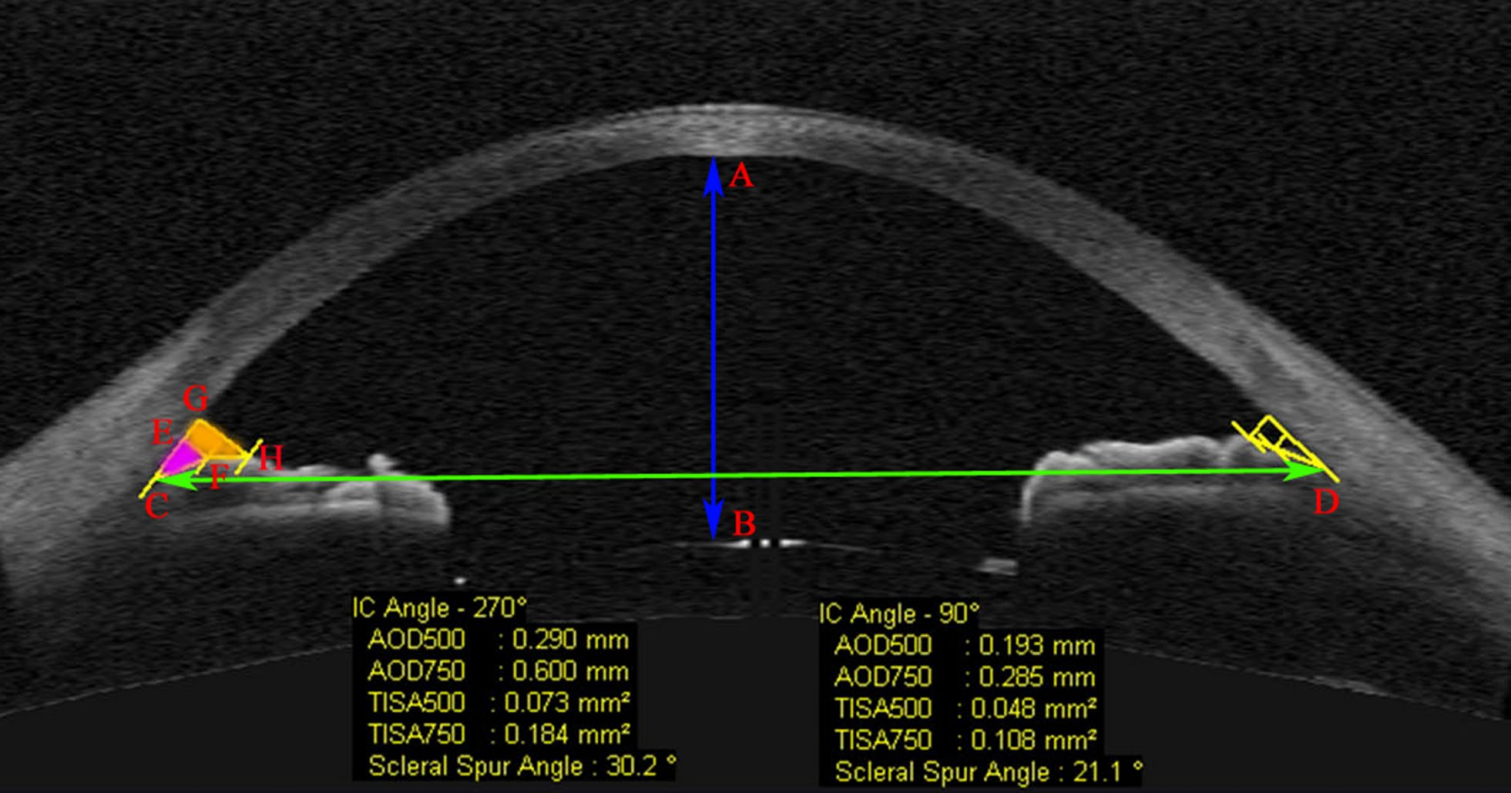
Anterior chamber morphological parameters recorded in this study

The Visante OCT software (version 2.0.1.88) was used for image analysis. A glaucoma specialist experienced in AS-OCT confirmed the quality of all images for each patient and manually identified the LSS and RSS in each image. The surfaces of the cornea, irides, and lens were delineated automatically by algorithm. Four anterior segment indices were defined and recorded: Anterior chamber width (ACW), anterior chamber depth (ACD), trabecular iris angle (TIA), angle opening distance (AOD), and trabecular iris space area (TISA) (Fig. 1). The definition of the parameters measured are listed in Table 1. AOD and TISA were measured at 500 and 750 µm of SS in 4 quadrants (0, 90, 180, and 270 angles). The mean of TIA, AOD and TISA (MTIA, MAOD and MTISA) at 500 (and 750 µm for MAOD and MTISA) for each eye was also calculated by mean of four quadrants values. All Images were analyzed by a single grader who was masked to the subjects’ clinical data. Additionally, all the images were graded a second time 2 weeks after initial evaluation by the same observe (who was masked to original measurements) to ensure consistency. The test–retest intraclass correlation coefficients were 93% and 95% for ACD and ACW, respectively.

**Table 1:** Definition of anterior segment parameters measured by anterior segment optical coherence tomography (AS-OCT) in this study

Parameter	Definition
Anterior chamber depth (ACD)	The axial distance from the corneal endothelium to the anterior lens surface. (AB arrow in Fig. 1)
Anterior chamber width (ACW)	The distance between the two scleral spurs. (CD arrow in Fig. 1)
Trabecular iris angle (TIA)	An angle measured with the apex in iris recess and the arms of the angle passing through a point on the trabecular meshwork 500 µm away from the SS and the corresponding perpendicular point on the iris. (The angle between CE and CF lines in Fig. 1)
Angle opening distance 500 and 750 µm (AOD500 and AOD750)	The length of a line drawn from the anterior iris to the corneal endothelium perpendicular to a line along the trabecular meshwork at 500 µm and 750 µm from the SS (EF and GH lines, respectively, in Fig. 1)
Trabecular iris space area 500 and 750 µm (TISA500 and TISA750)	An area covering 500 µm and 750 μm located in the area between the cornea and the iris. (Purple area and purple + orange areas, respectively, in Fig. 1)

### Surgery

After recording preoperative measurements, patients underwent pars plana vitrectomy without tamponade by an experienced vitreoretinal surgeon (A.KH). The surgical protocol was the same for all patients. In brief, a standard 3-port 23-gauge vitrectomy was done for each patient. Triamcinolone assisted posterior vitreous detachment and Brilliant Blue G assisted ILM peeling were done. The posterior hyaloid was separated as far as the equator and peripheral vitreous was removed as visible without scleral indentation. Therefore, vitreous base shaving with scleral depression was not performed for any eye. No intraoperative laser was done. None of the sclerotomies required suturing. Patients were treated with chloramphenicol 0.5% eyedrops every 6 h and betamethasone 1.0% every 4 h for 1 week. Betamethasone was tapered over the following 3 weeks. No cycloplegic eyedrop was prescribed.

After 1 and 6 months, all patients underwent slit-lamp bio microscopy of anterior chamber, Goldmann applanation tonometry, gonioscopy, and AS-OCT to determine post-operative anterior chamber morphologic parameters.

### Statistical analysis

Data were recorded as the mean, SD, median, and range. The Kolmogorov–Smirnov test and Q-Q plots were used to assess the normal distribution of quantitative variables. The mean value of quantitative variables was compared between the preoperative and the postoperative measurements using paired t-test due to normal distribution of values. All statistical analyses were performed using SPSS for Windows software (version 25.0, IBM Corp.) A P-value less than 0.05 was considered statistically significant.

## Results

30 eyes from 30 patients underwent PPV with 7 patients declining to complete post-operative follow-up requirements. 23 patients completed the measurements at months 1 and 6 of whom 13/23 (56%) were female. The mean age of participants was 56.4 ± 3.6 years of age. All patients were phakic.

The mean IOP of patients was 18.1 ± 1.1, 18.3 ± 1.1, and 18.1 ± 1.2 mmHg, at pre-operative, month 1 post-operative, and month 6 post-operative visits (*p* = 0.83). The differences in anterior segment measures on AS-OCT across visits is presented in Table 2 and did not reach statistical significance at any time-point.

**Table 2:** Anterior segment measures of studied patients

Parameter	Group			Diff	Diff 95% CI		P value *
	Preop	1 month	6 month		Lower	Upper	
ACD	3.19 ± 0.64	3.19 ± 0.65	3.42 ± 0.57	0.00 ± 0.23	− 0.10	0.09	0.90
				0.23 ± 0.53	0.00	0.46	0.06
ACW	11.78 ± 0.30	11.87 ± 0.39	11.85 ± 0.36	0.08 ± 0.23	− 0.01	0.19	0.08
				0.06 ± 0.27	− 0.04	0.18	0.24
TIA-0°	37.30 ± 15.42	38.70 ± 15.65	41.97 ± 5.05	1.40 ± 6.68	− 1.48	4.29	0.32
				4.67 ± 14.33	− 1.52	10.86	0.13
TIA-90°	31.37 ± 12.75	33.55 ± 12.59	32.53 ± 4.44	2.17 ± 8.63	− 1.56	5.90	0.24
				1.15 ± 11.79	− 3.94	6.26	0.64
TIA-180°	39.12 ± 13.67	40.93 ± 12.01	39.51 ± 6.66	1.81 ± 6.60	− 1.03	4.67	0.20
				0.39 ± 12.31	− 4.93	5.71	0.88
TIA-270°	34.05 ± 14.26	34.91 ± 12.14	35.56 ± 4.11	0.86 ± 7.57	− 2.41	4.13	0.59
				1.51 ± 14.21	-4.63	7.65	0.61
AOD500-0°	0.43 ± 0.25	0.45 ± 0.29	0.46 ± 0.13	0.02 ± 0.12	− 0.02	0.08	0.34
				0.03 ± 0.24	− 0.07	0.13	0.53
AOD500-90°	0.32 ± 0.16	0.36 ± 0.18	0.32 ± 0.09	0.03 ± 0.1	− 0.02	0.09	0.26
				0.00 ± 0.14	− 0.06	0.06	0.97
AOD500-180°	0.43 ± 0.18	0.46 ± 0.21	0.43 ± 0.18	0.03 ± 0.13	− 0.01	0.09	0.18
				0.00 ± 0.16	− 0.06	0.07	0.85
AOD500-270°	0.36 ± 0.20	0.36 ± 0.15	0.36 ± 0.09	0.00 ± 0.11	− 0.04	0.05	0.88
				0.00 ± 0.19	− 0.08	0.08	0.95
AOD750-0°	0.67 ± 0.33	0.68 ± 0.37	0.71 ± 0.17	0.00 ± 0.16	− 0.06	0.08	0.79
				0.04 ± 0.32	− 0.10	0.18	0.56
AOD750-90°	0.48 ± 0.22	0.53 ± 0.24	0.47 ± 0.12	0.05 ± 0.10	− 0.02	0.13	0.17
				− 0.00 ± 0.21	− 0.10	0.08	0.82
AOD750-180°	0.69 ± 0.29	0.71 ± 0.27	0.70 ± 0.30	0.02 ± 0.19	− 0.06	0.10	0.63
				0.00 ± 0.27	− 0.11	0.12	0.93
AOD750-270°	0.61 ± 0.32	0.60 ± 0.27	0.59 ± 0.15	− 0.01 ± 0.21	− 0.10	0.08	0.78
				− 0.02 ± 0.33	− 0.16	0.12	0.76
TISA500-0°	0.14 ± 0.08	0.15 ± 0.10	0.14 ± 0.04	0.01 ± 0.05	0.00	0.04	0.14
				0.00 ± 0.07	− 0.02	0.04	0.68
TISA500-90°	0.11 ± 0.05	0.11 ± 0.06	0.11 ± 0.03	0.00 ± 0.02	0.00	0.01	0.25
				0.00 ± 0.05	− 0.01	0.02	0.67
TISA500-180°	0.14 ± 0.06	0.16 ± 0.07	0.16 ± 0.07	0.02 ± 0.04	0.00	0.04	0.05
				0.01 ± 0.05	0.00	0.04	0.17
TISA500-270°	0.12 ± 0.06	0.11 ± 0.05	0.12 ± 0.04	− 0.00 ± 0.03	− 0.01	0.01	0.90
				0.00 ± 0.06	− 0.02	0.03	0.77
TISA750-0°	0.27 ± 0.14	0.30 ± 0.18	0.29 ± 0.08	0.02 ± 0.09	− 0.01	0.06	0.23
				0.01 ± 0.014	− 0.04	0.08	0.56
TISA750-90°	0.20 ± 0.09	0.22 ± 0.11	0.21 ± 0.05	0.02 ± 0.07	0.00	0.05	0.13
				0.01 ± 0.08	− 0.02	0.04	0.57
TISA750-180°	0.28 ± 0.12	0.31 ± 0.12	0.30 ± 0.12	0.02 ± 0.07	− 0.01	0.05	0.17
				0.01 ± 0.11	− 0.03	0.06	0.50
TISA750-270°	0.24 ± 0.12	0.24 ± 0.10	0.24 ± 0.06	− 0.00 ± 0.06	− 0.03	0.02	0.91
				0.00 ± 0.12	− 0.05	0.05	0.99

*ACD* anterior chamber depth, *ACW* anterior chamber width, *Diff* difference, *AOD* angle opening distance, *TIA* trabecular iris angle, *TISA* trabecular iris space area

**Fig. 2:**
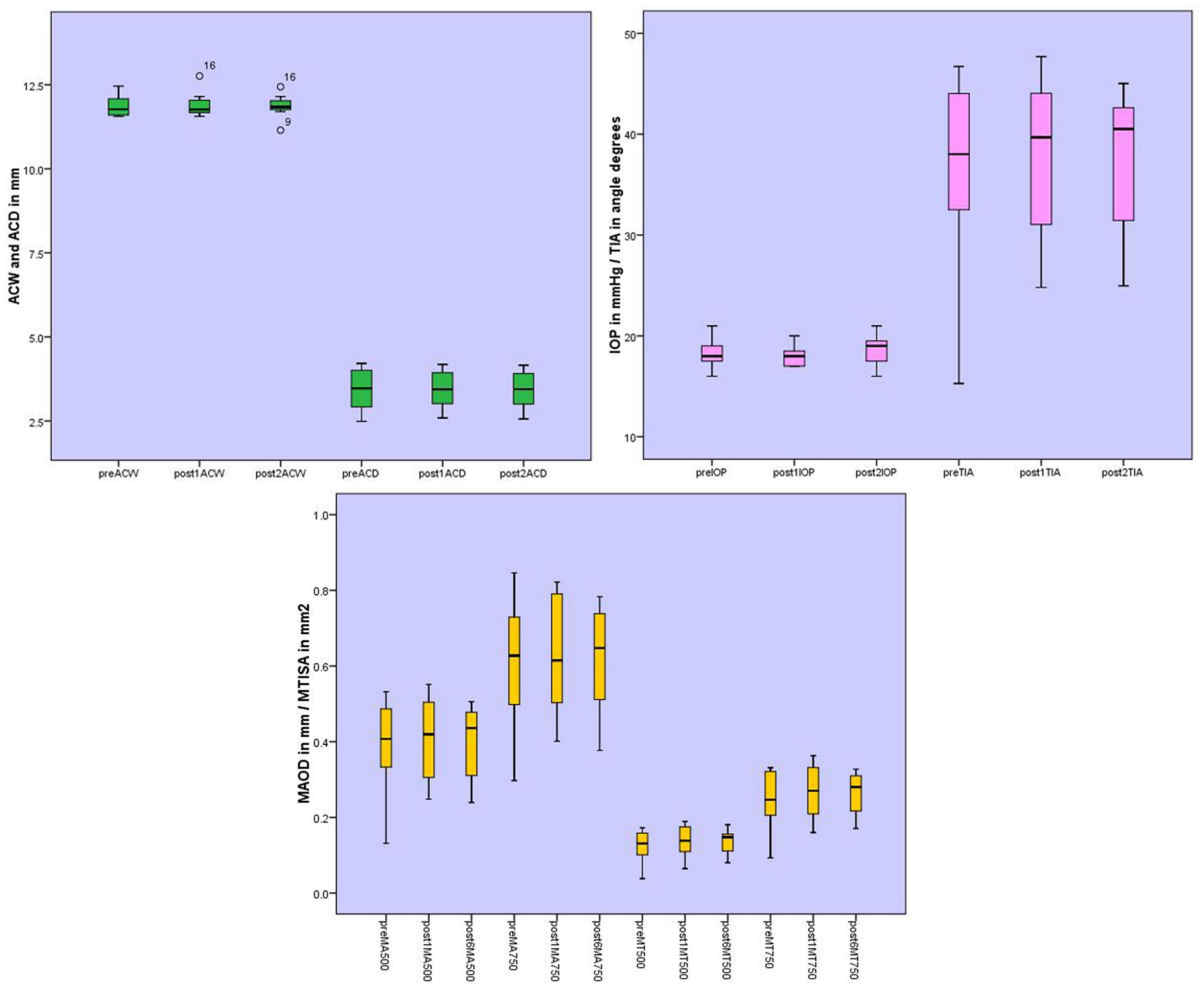
The box-plot of repeated measures of anterior chamber parameters and intraocular pressure. Measurements were done at preoperative, 1 month and 6 months. Upper left: Anterior chamber width (ACW) and Anterior chamber depth (ACD). Upper right: Intraocular pressure (IOP) and mean Trabecular iris angle (MTIA). Bottom: Mean Angle opening distance (MAOD) and Mean Trabecular iris space area (MTISA) at 500 and 750 µm of SS

Figure 2 presents the change in IOP and anterior segment measurements during the study period and demonstrates stability of all parameters (mean, lower quartile, and upper quartile) before and after PPV. Although differences were statistically insignificant, box and plot graphs showed that the range of most angle morphologic parameters including TIA, MAOD, and MTISA were reduced at postoperative measurements compared to preoperative measurements which was especially true for lower values (quartile group 1). Preoperative and postoperative measurements of ACD and IOP were similar across different quartiles of the parameters.

## Discussion

In this prospective study, anterior chamber morphology did not significantly change after PPV without intraocular tamponade: IOP, ACD, ACW, TIA, AOD, and TISA 1 month and 6 months post-operatively did not significantly differ from pre-operative measures.

Postoperative ocular hypertension occurs in 18–28% [1–3] of patients following in the first year after PPV [6]. Up to 30% of patients may develop secondary glaucoma within 2 years of PPV [6]. Fang et al. demonstrate that tamponade type was the only significant risk factor for elevated IOP after PPV whereas age, gender, preoperative IOP, refractive error, medical history, retinal diagnosis, and various combinations of PPV surgeries did not significantly correlate with the risk of IOP elevation post-operatively [4]. Nonetheless, other studies have suggested that even without tamponade or associated surgical procedures such as cataract extraction, there are instances of secondary glaucoma whether open-angle or closed-angle after pars plana vitrectomy [8, 17]. Koreen et al. found that 11.6% of vitrectomy patients had late-onset open-angle glaucoma after PPV whileHan et al. reported 41% of PPV patients had early-onset open-angle glaucoma and 18% early-onset closed-angle glaucoma after PPV. Siegfried et al. recently reported on the role of oxidative stress/damage to the trabecular meshwork following PPV which may contribute to IOP rise and increased risk of open angle glaucoma [18]. Prior investigations have found variable changes in AC morphology although differences in patient characteristics and measurement types make comparisons difficult (Table 3) [10–15].

**Table 3:** Review of studies evaluating anterior chamber changes following pars plana vitrectomy

Author, Year	Design	No. of eyes	Surgery	F/U (m)	Modality of measurement	Anterior chamber parameters
Tuklo, 2020	Prospective case series	88	complete PPV(44) and partial PPV(44)	3	Pentacam	ACD increased significantly in c-PPV but this increase was not significant in the p-PPV group ICA increased significantly in the c-PPV group but decreased in the p-PPV group No significant change in AL in each group
Ghomi, 2017	Prospective case series	7	PPV ± tamponade	6	UBM	ACD, CBD, CBW, AC angle did not change LAP was increased (3.9 vs. 4.1; p = 0.04)
Huang, 2016	Prospective case series	238	PPV + tamponade	12	SD-OCT	AL did not change ACD was decreased (3.07, 2.97, and 3.02 at preop, 6 months, and 12 months; p < 0.05)
Li, 2013	Prospective case series	29	Isolated PPV	3	A-scan	In PPV for vitreous hemorrhage: no change in ACD occurred In PPV for ERM: ACD was deeper compared to control eyes at pre-op and 1 week (p < 0.01), but there was no difference at 1 month and 3 months
Calik, 2013	Prospective cohort	44	PPV + SO (22) and PPV (22)	1	Pentacam	ACV and ACA did not change ACD was decreased in SO group (2.9 vs. 2.4; p < 0.05) while increased in sole PPV group (2.6 vs. 2.7; p < 0.05)
Neudorfer, 2011	Prospective cohort	28	Isolated PPV (13) and PPV + gas (15)	2 days	UBM	LAP did not significantly change ACD was decreased in gas group (3.1 vs. 2.7; p < 0.01) but not in isolated PPV group
Marigo, 2006	Prospective case series	20	Isolated PPV	3	UBM	ACD, AOD500, TCD, CBT, and SST did not change

*ACA* anterior chamber angular width, *ACD* anterior chamber depth, *AL* axial length, *AOD* angle opening distance, *CBD* ciliary body depth, *CBW* ciliary body width, *CBT* ciliary body thickness, *ICA* iridocorneal angle, *ERM* epiretinal membrane, *LAP* lens anterior posterior diameter, *PPV* pars plana vitrectomy, *c-PPV* complete PPV, *p_PPV* Partial PPV, *SO* silicone oil, *SST* supra ciliary thickness, *TCD* trabeculocilliary distance, *UBM* ultrasound biomicroscopy

Most investigations have not found significant changes in key anatomic parameters including angle opening distance, ciliary body depth, width, and thickness, trabeculocilliary distance, and supra ciliary thickness. Lens thickness was increased following PPV in Ghomi et al.’s study [14] but not in Neudorfer et al.’s investigation [10]. Calik et al. [15] Neudorfer et al. [10] and Huang et al. [13] all demonstrated that Anterior segment depth (ACD) decreased following PPV with tamponade. Changes observed following PPV without tamponade have been inconsistent with Calik et al. demonstrating an increase in ACD with pentacam [15], Neudorfer et al. finding no change in ACD with UBM measurment [10], and Li et al. finding no significant change in ACD among patients with vitreous hemorrhage or ERM [12]. Although Li et al. showed a non-significant increase in ACD in 6 patients who underwent PPV for ERM after 3 months but the inter-eye ACD difference was decreased significantly which was due to a larger increase in ACD measurement in fellow non-vitrectomized eyes during follow ups. This pattern may be attributed to their measurement method as they used A-scan ultrasound which was more operator dependent. [12] Indeed, these variations might be due to the different imaging modalities in addition to inter or intra-observer biases. In addition, Toklu et al. revealed that not removing the vitreous base with scleral indentation in PPV surgeries (partial PPV) in contrast to complete PPV, may create a more stable anterior chamber and prevent the reduction in ACD [19].

Most prior studies that evaluated angle morphology after PPV used UBM [3, 10, 11] which is a real-time imaging modality that uses high-frequency (40–100 MHz) ultrasound with a penetration depth of 5 mm which provides high-resolution and detailed images of AC [20]. Its lateral and axial resolutions are 50 and 25 µm, respectively. UBM can be particularly helpful in eyes with opaque media [20] Nevertheless, it has several limitations including the need for a highly skilled operator, not only to avoid inadvertent pressure on the eyecup (that may influence the angle configuration), but also to localize the anatomical landmarks and provide the best image for measuring the distances [16]. AS-OCT is a light-based imaging modality which carries several advantages [16]. The resolution of AS-OCT is approximately 15 µm which provides higher resolution images and excellent visualization of angle structures. Additionally, AS-OCT is not as technically difficulty as UBM and can be done with minimal expertise that can reduce the possibility of intra and inter-observer biases [16]. One limitation of AS-OCT is its inability to visualize the entire ciliary body due to its limited penetration. In a comparative study of angle visualization between UBM and AS-OCT, UBM produced better visualization of the ciliary body and angle recess while AS-OCT provided excellent delineation of angle structures, iris surface, and distinct critical landmarks such as the scleral spur [16]. Although both imaging modalities had similar reproducibility of various anterior segment parameters, comparisons between the two imaging modalities did produce some small statistically significant differnees with UBM tending to give smaller measurements [16].

In the present study, quantitative measurement of the AC angle was done with AS-OCT. ACD and ACW were not different between preoperative and postoperative measurements. TIA, AOD and TISA at 500 µm (and 750 µm for AOD and TISA) anterior to the scleral spur were calculated in four quadrants and no significant changes were observed over the course of follow-up. Consistent with previous studies (Table 2), IOP and anterior chamber anatomy remained stable after PPV without tamponade (Fig. 2). This is also consistent with clinical findings reported by Fang et al. that reported no incidence of IOP rise during one year follow up of patients underwent PPV without tamponade [4]. There are no reports of late-onset (long-term) closed-angle glaucoma following isolated PPV and this may be because of the preserved anatomic structures following standard PPV.

The box-plot graphs of angle morphologic parameters showed that although the central tendency for these variables were consistent across visits, there was a tendency for the dispersion of values to be less postoperatively. The range of TIA, MAOD, and MTISA parameters were all reduced postoperatively especially at first quartile. Although, there was no change following PPV in the morphologic parameters of angles of 30 to 45 degrees, anterior chambers more shallow than 30 degrees tend to become deeper compared to preoperative status. More detailed subgroup analysis was not performed due to small sample sizes. For ACW, ACD, and IOP, dispersion of postoperative values was consistent compared to pre-operatively. Importantly, none of the patients in this study underwent intraoperative laser which could have affected angle configuration via ciliary body edema.

The main limitation of this study was its sample size which limited the statistical power to detect small differences. Further studies are necessary to categorize changes in anterior chamber morphology based on preoperative anterior chamber configuration. Also, the lack of control eyes makes it difficult to generalize the results. The presence of ERMs may be a confounding factor; however, there are no known correlations between angle position and the presence of ERMs.

This is the first study evaluating quantitative parameters of the AC angle before and after isolated PPV. These results suggest that anterior chamber morphology may not change significantly after PPV without intraocular tamponade for eyes with ERM. To verify these results, larger comparative studies needed to be conducted.

## Data Availability

The datasets during and/or analysed during the current study available from the corresponding author on reasonable request.
